# Association of evening chronotype, sleep quality and cognitive impairment in patients with major depressive disorder

**DOI:** 10.3389/fpsyt.2024.1494032

**Published:** 2024-11-18

**Authors:** Li Wang, Yingchao Huo, Lihua Yao, Nan Zhang, Simeng Ma, Zhaowen Nie, Wei Wang, Enqi Zhou, Shunsheng Xu, Shenhong Weng, Dan Xiang, Maolin Hu, Zhongchun Liu

**Affiliations:** ^1^ Country Department of Psychiatry, Renmin Hospital of Wuhan University, Wuhan, China; ^2^ Taikang Center for Life and Medical Sciences, Wuhan University, Wuhan, China

**Keywords:** major depressive disorder, cognitive impairment, evening chronotype, sleep quality, digit symbol substitution test

## Abstract

**Objective:**

This study aimed to investigate the independent or synergistic effects of evening chronotype and poor sleep quality on cognitive impairment in patients with major depressive disorder (MDD).

**Methods:**

A cross-sectional study was conducted on 249 individuals diagnosed with MDD, recruited from the Mental Health Center of Renmin Hospital of Wuhan University. Chronotype preference was assessed using the reduced Horne and Ostberg Morningness - Eveningness Questionnaire (rMEQ), while sleep quality was evaluated using the Pittsburgh Sleep Quality Index (PSQI). Cognitive function was evaluated through the Digit Symbol Substitution Test (DSST), defining impairment as a DSST score ≤ 56 (the lowest quartile of the cohort). Univariate analysis and logistic regression models were employed to explore the factors associated with cognitive impairment, focusing on the potential interactive effects of evening chronotype and poor sleep quality.

**Results:**

Of the 249 subjects recruited, about 41% were classified as evening chronotype. These individuals exhibited poorer sleep quality and more severe depressive symptoms compared to non-evening chronotype (p < 0.01). Univariate analysis revealed that first episode status, Hamilton Depression Rating Scale (HAMD-17) scores, evening chronotype, and poor sleep quality were significantly associated with cognitive impairment (p < 0.05). Multivariate logistic regression analysis further demonstrated that the co-existence of evening chronotype and poor sleep quality significantly increased the likelihood of cognitive impairment (adjusted odds ratio [AdjOR] = 2.65, 95% confidence interval [CI] = 1.09–6.45, p < 0.05).

**Conclusion:**

Our findings suggest that evening chronotype, poor sleep quality, and their interaction are important contributors to cognitive impairment in patients with MDD, alongside the severity of depression and first episode status. These results emphasize the need for integrated approaches targeting circadian rhythm disruptions and sleep disturbances in the treatment of cognitive dysfunction in MDD.

## 1 Introduction

Major depressive disorder (MDD) is a disabling psychiatric condition that has been associated with impairments across emotional, functional, and cognitive deficits, along with executive functions, language, and working memory dysfunctions (1). Clinically, MDD patients frequently present with cognitive complaints, mainly encompassing difficulties in thinking, concentration, and decision-making. Cognitive dysfunction in MDD spans various areas such as memory, executive function, processing speed, and attention, with a notable impairment in executive functions (2, 3). These cognitive impairments significantly influence treatment outcomes, rehabilitation processes, quality of life, and social activities of MDD patients, and are particularly associated with a poorer response to medication (4) and psychosocial dysfunction (5). In patients with MDD, circadian disruption and poor sleep quality are prevalent, exacerbating depressive symptoms and cognitive dysfunction (6, 7). Nonetheless, limited studies have evaluated the impacts of circadian rhythm and sleep disturbances on cognitive dysfunction in MDD.

Patients with MDD commonly experience altered circadian rhythms, sleep disturbances, and mood changes with circadian cycle. Individual circadian preference, reflecting behavioral preferences for sleep-wake and daily activities, varies significantly among individuals. Chronotypes are commonly classified as morningness, intermediate and eveningness preferences. Irregularities in circadian rhythms and sleep patterns have been frequently observed not just during acute episodes but also during the prodromal and remission phases of depression (8). Even minor shifts in the circadian phase of sleep can profoundly affect sleep quality and duration (9–11). Disturbances in circadian rhythms and sleep are increasingly recognized as playing crucial roles in the pathophysiology of mood disorders (12).

The severity of depression in MDD patients has been linked to circadian misalignment (13). The evening chronotype could be proposed as a potential vulnerability factor for depression, while the morning chronotype may be protective (14, 15). Evening preference has been reported to be associated with an increased risk of MDD and various other psychiatric symptoms or disorders, including poor sleep, anxiety, bipolar disorder and seasonal affective disorder (16, 17). Notably, recent research indicates that in patients with fibromyalgia, higher eveningness, greater severity of diurnal dysrhythmia, lower wakeability, and poorer sleep quality predict a lack of response to SNRI antidepressants (18). Additionally, poor sleep quality has been shown to exacerbate affective symptoms, foster negative cognitive biases, and diminish sustained attention to non-affective stimuli (15, 19). Importantly, poor sleep quality has been identified as a significant predictor for the onset of major depression (20). Sleep quality in patients with MDD may mediate a significant association between evening preference and depressive symptoms (15).

There is a potentially complex relationship between cognitive impairment, sleep disturbance, and chronotype. A meta-analysis reported that sleep disorders were associated with poorer cognitive performance in general and multiple specific cognitive domains (21). Both severely impaired sleep quality and evening preference contribute to cognitive impairment in MDD patients (22). Furthermore, studies have revealed a link between circadian preference and cognitive deficits. For example, patients with delayed sleep-wake phase disorder often show pronounced attention problems, especially in the morning and when forced to wake early (23). Additionally, depressed patients with evening preference self-report more domains of cognitive impairments compared to those with morning and intermediate types (24). Historical studies in this area did not systematically account for variables like sleep quality or current mood states together, which could influence cognitive performance in MDD. Sleep disturbances and evening preference are associated with non-remission of major depression (25). However, the impact of sleep quality and whether sleep quality and chronotype preferences on cognitive impairment in MDD was poorly reported.

Currently cognitive assessment tools are often time-consuming and intricate. Variability in research findings regarding cognitive performance in MDD patients can be attributed to differing test timings, the specific neuropsychological tasks employed, and diverse study designs. Although a range of cognitive measures are utilized in randomized controlled trials for MDD therapeutic interventions, the Digit Symbol Substitution Test (DSST) stands out as the only cognitive endpoint available as a test for cognitive dysfunction in the studies assessed (26). DSST is a ‘pencil and paper’ cognitive test that evaluates several of the most impaired aspects of cognitive function in MDD, such as components of executive function, processing speed, attention, and working memory.

Therefore, rhythm and sleep disturbances are prevalent in MDD, while both circadian rhythm disruptions and poor sleep quality are involved in the onset and outcome of depression, their independent or synergistic effects on cognitive deficits have been reported rarely. The study aims to characterize the clinical features of depressed patients based on the eveningness - morningness chronotype. Furthermore, we investigate the association between self-reported diurnal preference, sleep quality and cognitive performance as measured by the DSST in patients with depression.

## 2 Methods

### 2.1 Participants

This study was part of a larger Chinese “Early-warning System and Comprehensive Intervention for Depression” (ESCID) project from April 2019 to July 2020. The study targeted outpatients and inpatients aged 18 - 39 years with Major Depressive Disorder (MDD) from the Mental Health Center of Renmin Hospital of Wuhan University. Diagnosis was confirmed by two experienced psychiatrists using the Mini-International Neuropsychiatric Interview (27). All clinicians evaluating the scales were all strictly trained for consistency. Participants met the diagnostic criteria for major depressive disorder of the Diagnostic and Statistical Manual of Mental Disorders Fifth Edition (DSM-5), underwent questionnaire and routine laboratory screening. Patients with a history of bipolar disorders, schizophrenia spectrum disorders, intellectual disability, dementia, and alcohol or substance misuse/dependence were excluded. Shift workers and subjects who were unable to provide valid informed consent were excluded. Additionally, those with severe physical illness or craniocerebral trauma that rendered them unable to complete the clinical interview were also excluded.

All participants were informed of the purpose of the study and agreed to participate before the survey, and the Ethics Committee of Renmin Hospital of Wuhan University approved the study protocol (approval number: WDRY2020-K191).

### 2.2 Socio-demographical and clinical characteristics

Age, gender, ethnicity, place of residence, marital status, educational status, employment status, body mass index (BMI), and age-on-set were recorded. The ethnicities were all Chinese, divided into Han group and non-Han group (including Hui, Yao, Mongol, Tujia, Buyi, etc.). Place of residence was categorized as urban and rural. Marital status was categorized as married, single/divorced. Educational status was grouped into high school graduate and below, or college graduate and higher. Employment status was categorized as employed and unemployed.

### 2.3 Severity of depressive symptoms and current mood

HAMD-17 is a 17-item replacement scale used to assess the severity of depressive symptom (28). Each question has a score of 0 - 2 or 0 - 4, with a total score of 0 - 52. Higher total scores indicate more severe depressive symptoms. The scores are interpreted as follows: 0 - 7 for no depressive symptoms, 8 - 17 for mild depressive symptoms, 18 - 24 for moderate depressive symptoms, and 25 - 52 for severe depressive symptoms.

### 2.4 Measure of chronotype preference

The reduced version of Horne and Ostberg Morningness Eveningness Questionnaire (rMEQ) was used to measure chronotype preference (29). The questionnaire yields scores ranging from 4 to 24, with common reference categories to the following cut - offs: evening type (score < 12), intermediate type (score 12 - 17) and morning type (score > 17). The rMEQ has been validated for use in ethnic Chinese individuals and demonstrates robust psychometric properties (30). Due to the limited number of morning-type samples, we divided the samples into two groups: evening type and non-evening type.

### 2.5 Sleep quality assessment

The Pittsburgh Sleep Quality Index (PSQI) is a self-rated questionnaire that assesses subject sleep quality and disturbances over a one-month period (31). The 18 items of the PSQI form a seven-component score ranging from 0 to 3, which can be summarized as a general score. The total score ranges from 0 - 21, with higher scores indicating worse sleep quality. For subgroup analysis, a PSQI total score > 10 was defined as significantly impaired sleep quality, referring to previous literature (22).

### 2.6 Cognitive impairment evaluation

Cognitive impairment was defined as a DSST score falling with lowest quartile. The score is the total number of correct symbols drawn within 2 minutes, with one point given for a correct response and a maximum score of 133. Since there is no well-defined threshold of DSST score for detecting cognitive impairment, the lowest unweighted quartile in the study population (DSST ≤ 56) was used to define cognitive impairment or low cognitive function, consistent with methods previously used (32, 33). Subjects with DSST scores > 56 were considered not to be cognitively impaired.

### 2.7 Statistical analysis

Continuous variables were expressed as mean (M) ± standard deviation (SD), while categorical variables were expressed as percentages. The normality of data distributions was assessed using the Kolmogorov-Smirnov test. Differences between groups for normally distributed variables were analyzed using the t-test, while the chi-squared and Kruskal-Wallis tests were employed for categorical variables and non-normally distributed variables, respectively. Variables demonstrating significance (p < 0.05) in the univariate analysis were further evaluated using logistic regression models. Binary logistic regression models were established to explore the effects of both evening chronotype and impaired sleep quality on cognitive impairment, sequentially adjusting for variables such as severity of depression (HAMD-17 total scores > 17) and first episode status (Model 2 and 3), as well as age and gender (Model 4). The strength of the association was measured by adjusted odds ratios (Adj OR) with 95% confidence intervals (CI). A p value of less than 0.05 was considered as statistical significance. SPSS Statistic 26.0 was used for all the data analyses.

## 3 Results

### 3.1 Study population characteristics by chronotype

A total of 249 participants were eligible for the present study and were grouped by diurnal preference based on a split of the rMEQ scores. They were labeled as evening preference and non-evening preference, respectively. **Table 1** lists a detailed comparison of the socio-demographic and clinical characteristics between two groups. Both groups were comparable regarding age, gender, ethnicity, BMI, place of residence, marital status, education, and employment status. However, the evening preference group exhibited significantly higher HAMD-17 total scores, poorer sleep quality, and lower DSST scores compared to the non-evening preference group.

**Table 1:** Socio-demographic and clinical characteristics of patients with MDD by chronotype.

Characteristics	Total	Evening type	Non-Evening Type	P
	(n = 249)	(n = 102)	(n = 147)	
Age, yr, mean (SD)		21.8 (3.44)	22.6(4.18)	0.114
Age-at-onset, yr, mean (SD)		19.0 (4.31)	19.8(5.13)	0.366
First-episode, n (%)	161 (64.7)	63 (61.8)	98 (66.7)	0.426
Gender				
Female, n (%)	191 (76.7)	73(71.6)	118 (80.3)	0.110
Male, n (%)	58 (23.3)	29(28.4)	29 (19.7)	
Han nationality, n (%)	232 (93.2)	94(92.2)	138 (93.9)	0.597
BMI		21.1(5.09)	20.9(4.02)	0.743
Resident				
Urban, n (%)	220 (88.4)	89 (87.3)	131 (89.1)	0.653
Rural, n (%)	29 (11.6)	13 (12.7)	16 (10.9)	
Marital status				
Married, n (%)	13 (5.2)	4 (3.9)	9 (6.1)	0.443
single or divorced, n (%)	236 (94.8)	98 (96.1)	138 (93.9)	
Educational status				
In college and below, n (%)	161 (64.7)	66 (64.7)	95 (64.6)	0.990
Graduate from college or higher, n (%)	88 (35.3)	36 (35.3)	52 (35.4)	
Employment				
Employed, n (%)	46 (18.5)	18 (17.6)	28 (19.0)	0.779
Unemployed, n (%)	203(81.5)	84 (82.4)	119(81.0)	
HAMD-17 score, mean (SD)		21.3 (5.93)	16.1 (7.67)	< 0.001
PSQI score, mean (SD)		11.8(3 42)	9.0 (4.07)	< 0.001
DSST score, mean (SD)		60.2 (13.41)	66.8 (11.71)	< 0.001

MDD, Major depressive disorder; HAMD, Hamilton depression scale; PSQI, Pittsburgh sleep quality index; DSST, Digit symbol substitution test.

### 3.2 Participant characteristics by cognitive function status

The characteristics of the study participants, stratified by cognitive function status, were detailed in **Table 2**. Of the participants, 67 (26.9%) were defined as having cognitive impairment with an unweighted DSST score ≤ 56. Compared to subjects without cognitive impairment, those with cognitive impairment had significantly higher scores on the depression scale, poorer sleep quality, and a greater likelihood of being classified as having an evening chronotype.

**Table 2:** Comparisons of demographic features, sleep characteristics and psychological correlates according to cognitive impairment.

Characteristics	Total	Cognitive impairment	Non-Cognitive impairment	P
	(n = 249)	(n = 67)	(n = 182)	
Age, yr, mean (SD)		22.7 (4.73)	22.1 (3.56)	0.967
Age(age-at-onset), yr, mean (SD)		19.5 (5.40)	19.4 (4.60)	0.636
First-episode, n (%)	161(64.7)	36 (53.7)	125 (68.7)	0.029
Gender				
Female, n (%)	191 (76.7)	48 (71.6)	143 (78.6)	0.251
Male, n (%)	58 (23.3)	19 (28.4)	39 (21.4)	
Han nationality, n (%)	232 (93.2)	63 (94.0)	169 (92.9)	0.745
BMI		22.1(7.01)	20.6(3.00)	0.862
Resident				
Urban, n (%)	220 (88.4)	57 (85.1)	163 (89.6)	0.328
Rural, n (%)	29 (11.6)	10 (14.9)	19 (10.4)	
Marital status				
Married, n (%)	13 (5.2)	6 (9.0)	7 (3.8)	0.108
single or divorced, n (%)	236 (94.8)	61 (91.0)	175 (96.2)	
Educational status				
In college and below, n (%)	161 (64.7)	46 (68.7)	115 (63.2)	0.423
Graduate from college or higher, n (%)	88 (35.3)	21 (31.3)	67 (36.8)	
Employment				
Employed, n (%)	46 (18.5)	13 (19.4)	33 (18.1)	0.819
Unemployed, n (%)	203 (81.5)	54 (80.6)	149 (81.9)	
HAMD-17 score, mean (SD)		20.8 (7.55)	17.3 (7.21)	< 0.001
PSQI score, mean (SD)		11.1 (4.17)	9.8 (3.97)	0.027
rMEQ				
E-type, n (%)	102 (41.0)	38 (56.7)	64 (35.2)	0.002
non-E-type, n (%)	147(59.0)	29 (43.3)	118 (64.8)	

MDD, Major depressive disorder; BMI, Body mass index; HAMD, Hamilton depression scale; PSQI, Pittsburgh sleep quality index; rMEQ, reduced version of the Morningness–Eveningness questionnaire.

### 3.3 Current depression severity and cognitive functioning in patients with MDD: subgroup analysis of diurnal preference and sleep quality

**Figure 1:**
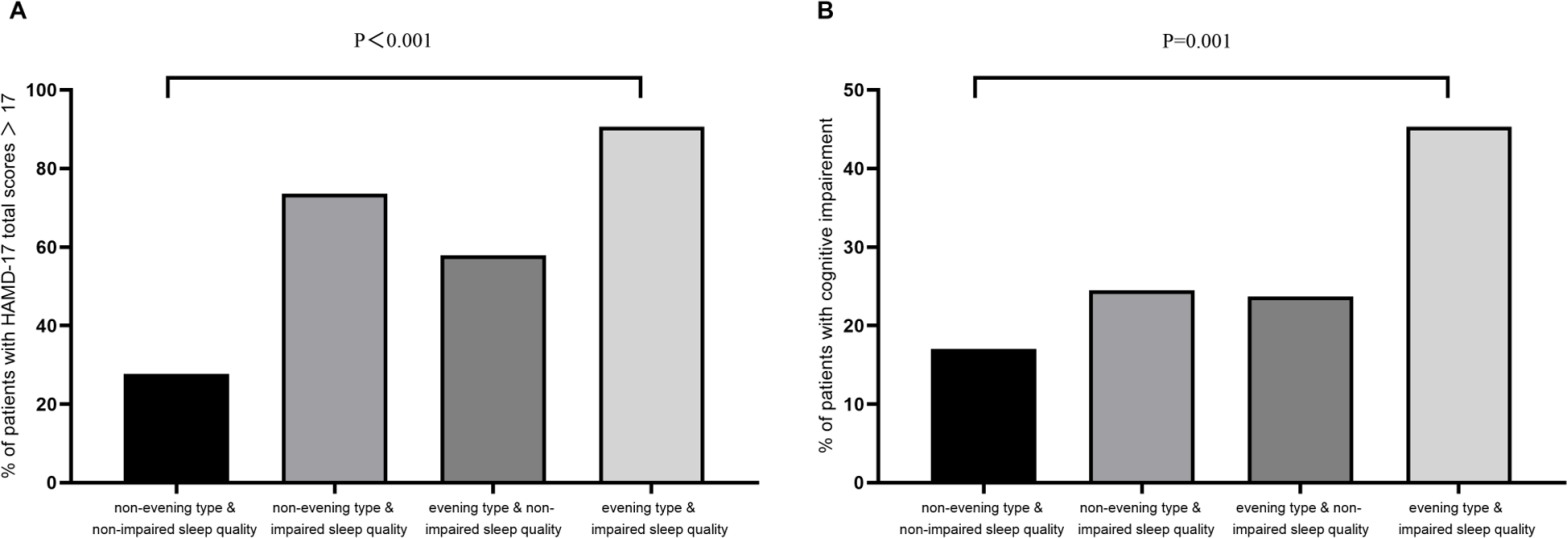
Subgroup analysis of diurnal preference and sleep quality. **(A)** Presence of sleep quality and evening type in relation to moderate to severe depression measured by HAMD-17 in MDD; **(B)** Presence of sleep quality and evening type in relation to impaired cognition measured by DSST in MDD. MDD, Major depressive disorder; HAMD, Hamilton depression scale; DSST, Digit symbol substitution test.

In order to investigate the association of diurnal preference and sleep quality with cognitive performance in MDD patients, we carried out a subgroup analysis by further dividing the sample according to the severity of sleep quality impairment (PSQI ≤ 10vs. > 10). The sample was categorized into four groups: “non-evening type with non-impaired sleep quality” (n=94), “non-evening type with impaired sleep quality” (n=53), “evening type with non-impaired sleep quality” (n=38), and “evening type with impaired sleep quality” (n=64). The proportion of patients with more severe depression (HAMD-17 total scores > 17 points) was generally higher among those MDD patients with impaired sleep quality regardless of diurnal preference (“non-evening type + non-impaired sleep quality” vs “non-evening type + impaired sleep quality” vs “evening type + non-impaired sleep quality” vs “evening type + impaired sleep quality”: 27.7% vs 73.6% vs 57.9% vs 90.6%; linear by linear association: χ² = 66.9, p < 0.001; **Figure 1A**). Similarly, the prevalence of relatively cognitive impairment, as measured by the DSST, was 17.0%, 24.5%, 23.7%, and 45.3% in the four groups, respectively (linear by linear association: χ² = 16.0, p = 0.001; **Figure 1B**).

### 3.4 The associations of evening type and impaired sleep quality with cognitive impairment in MDD

As shown in **Table 3**, compared with “non-impaired sleep quality and non-evening type” group, individuals categorized as “evening type and impaired sleep quality” demonstrated an increased risk of cognitive impairment (OR = 4.03, 95% CI = 1.95-8.37, p < 0.001). After further adjustment for variables, including the severity of depression, first episode, age, gender, education, and BMI, the correlation was relatively weakened, but the results were still statistically significant (Model 4: AdjOR = 2.65, 95% CI = 1.09-6.45, p < 0.05). Conversely, no significant differences in cognitive impairment were observed between the other two groups and the reference group (p > 0.05). Additionally, it is worth noting that more severe depression, as measured by HAMD-17, was independently associated with cognitive impairment in all adjustment models (Model 4: AdjOR = 2.48, 95% CI = 1.15–5.39, p = 0.021). Furthermore, compared with first episode depressive disorder, patients with recurrent depression had a higher risk of more severe cognitive impairment in both models 3 and 4 (Model 4: AdjOR = 1.97, 95% CI = 1.05–3.67, p = 0.034).

**Table 3:** Association of evening preference and sleep quality and cognitive impairment measured in MDD.

Variable	Cognitive impairment							
	Model 1		Model 2^a^		Model 3^b^		Model 4^c^	
	OR (95% CI)	P	OR (95% CI)	P	OR (95% CI)	P	OR (95% CI)	P
non-evening type & non-impaired sleep quality	1		1		1		1	
impaired sleep quality only	1.58(0.69-3.62)	0.274	1.07(0.44-2.62)	0.880	1.06(0.43-2.58)	0.905	1.05(0.42-2.67)	0.912
evening type only	1.51(0.60-3.80)	0.378	1.16(0.45-3.04)	0.760	1.13(0.43-2.98)	0.800	1.30(0.48-3.55)	0.610
evening type & impaired sleep quality	4.03(1.95-8.37)	< 0.001	2.41(1.15-5.06)	0.037	2.34(1.01-5.43)	0.049	2.65(1.09-6.45)	0.032

Cognitive impairment = 1, non-Cognitive impairment = 0. CI, confidence interval; OR, odds ratio; MDD, Major depressive disorder; BMI, Body mass index; HAMD, Hamilton depression scale.

^a^ Adjusted for HAMD-17 total scores > 17; ^b^ Adjusted for HAMD-17 total scores > 17 and first - episode; ^c^ Adjusted for age, gender, BMI, education, HAMD-17 total scores > 17 and first - episode.

## 4 Discussion

The current study investigated the association of chronotype and poor sleep quality with cognitive impairment in MDD. Our results revealed a high prevalence of severe depressive symptoms and cognitive impairment among those with evening chronotype and poor sleep quality. Specifically, we observed that evening preference and poor sleep quality could contribute to cognitive dysfunction, while the co-existence of these two conditions could confer a greater risk in MDD.

In line with previous research (15, 34), we found that evening preference was common among patients with MDD, especially those with concomitant poor sleep quality. Approximately 41% of the participants in the study sample were classified as evening type. They demonstrated poorer sleep quality when compared to those who were non-evening types. The finding was consistent with existing literature, which suggests that individuals with evening chronotypes have been found to show greater sleep-wake irregularities, wakefulness distress and greater sensitivity to poor sleep quality than those with other chronotypes (11). Moreover, evening-type patients with sleep disorders have a higher probability of irregular sleep habits and sleep-related dysfunctional cognitions, which in turn may perpetuate evening-type sleep disorders (35, 36). The high prevalence of evening preference comorbid sleep disturbance emphasizes the importance of considering circadian factors when assessing and managing sleep and mood problems in patients with MDD.

Our study did not observe significant differences in age, gender and other variables among the depressed individuals. Similarly, earlier research has found no substantial difference between sociodemographic characteristics including education, gender, marital status, income, and sleep quality. These demographic variables have no significant influence on the direct and indirect relationship between sleep quality-mediated chronotype preference and depressive symptoms in MDD (15).

Recent research indicates that the cognitive performance of Chinese patients with MDD is influenced by a range of clinical characteristics, including age, age of onset, depression severity, years of education, and sleep disturbances (37). Contrary to these findings, our study did not identify the influence of sociodemographic factors such as age, age of onset and educational attainment on neurocognitive impairment in MDD. Our findings do suggest, though, that cognitive impairment may be more related to the recurrence of MDD (38). The variability in results across different studies may be attributed to the diversity of the samples of patients with MDD, which can vary in terms of current depressive symptom severity, psychotropic medication use, age, comorbidities, and assessment tools employed (3, 37, 39). There is evidence that higher educational attainment can serve as a protective factor against cognitive dysfunction in MDD and might even compensate for cognitive deficits that have already occurred (40, 41). In our study, the participants were generally young and mostly students or working individuals, which might have partially compensated for their cognitive impairment. This underscores the importance of considering educational levels in the clinical evaluation of MDD patients, as higher levels of education appear to be associated with better cognitive function, regardless of depression severity (42).

Existing evidence suggests that improving cognitive function with the use of antidepressants is often associated with alleviations in the severity of depressive symptoms (43). Our study similarly found that cognitive impairment was independently related to the severity of major depressive disorder, aligning with meta-analyses (44) reporting that depression severity was associated with cognitive performance in episodic memory, executive function, and processing speed, explaining psychomotor slowing as a feature of more severe depression that may lead to impairments in the cognitive aspects of patients with MDD. It is important to note that MDD patients often show significant impairments in tasks requiring episodic memory and mental flexibility compared to healthy adults and those with mild depression, the latter two groups exhibiting similar neurocognitive performance (45).

However, inconsistencies exist in the literature regarding the extent to which depression severity impacts cognitive performance. McDermott (44) highlighted that these discrepancies might be due to the varying efficacy of neuropsychological tests in the cognitive field used, as well as differences in sample characteristics among studies. The cognitive deficits observed in learning and memory, executive function, processing speed, and attention and concentration in the DSST tests are frequently documented in MDD patients undergoing major depressive episodes. The area of cognitive dysfunction is broad in MDD and is associated with depressed mood, advocating that combined conventional antidepressant treatments to improve depressed mood can help facilitate cognitive improvements so far (46). However, improvements in depressive mood do not necessarily translate into cognitive enhancements, reinforcing the notion that the depression severity remains a significant risk factor for cognitive-related dysfunction in MDD.

While numerous studies have documented the effects of sleep disturbance and evening preference on depressive symptoms and cognitive function, few have investigated these factors simultaneously. In our study, a combination of clinical interviews and objective measures was employed to confirm the diagnosis of sleep and depressive disorders. Our findings revealed that cognitive performance was negatively influenced by evening preference, poor sleep quality and the severity of depressive symptoms, which was supported by prior research suggesting circadian rhythms and sleep quality affect cognition regardless of current depressive mood (22). The study further demonstrated that the co-occurrence of evening preference and impaired sleep quality independently elevates the risk of cognitive impairment in MDD. This association remained significant even after adjusting for potential confounders such as age and sex, further validating the combined impact of circadian preference and sleep on neuropsychological outcomes in MDD.

Furthermore, existing evidence suggests that the multifactorial nature of sleep disorders can significantly influence overall life satisfaction. For instance, studies have shown a clear relationship between pain severity, life satisfaction, and sleep quality in individuals with temporomandibular disorders (47). Additionally, a polysomnographic study has revealed that sleep quality is intricately linked to orofacial pain and headache complaints (48). Such findings indicate that psychological factors, such as depression, can exacerbate sleep disturbances, thereby affecting life satisfaction. Therefore, improving sleep quality may alleviate cognitive deficits in patients with major depressive disorder and enhance their overall health and life satisfaction, reinforcing the importance of integrated treatment strategies that address both sleep and emotional health.

Poor sleep quality, a prevalent biological symptom of MDD, was found to be intricately connected with circadian preference, thereby impacting neuropsychological performance. This study corroborated earlier findings indicating that both evening chronotype and poor sleep quality were independently and directly associated with higher depression severity in patients with depressive syndromes (49). Moreover, both sleep disturbances and evening preference were strongly associated with non-remission of MDD (25), implying that sleep quality and circadian rhythms might play unique roles in the development and maintenance of depression. The complex interrelations among circadian preference, sleep disturbances, severity of depression, and cognition highlight the need for further longitudinal and interventional studies to elucidate their intricate dynamics and causality. Severe sleep disturbances combined with an evening preference may act as a “double whammy” on brain function, exacerbating the cognitive deficits observed in MDD patients. Therefore, comprehensive systematic identification and assessment of sleep and circadian preferences should be integral to the daily clinical management of depression, as this may help and accelerate the functional recovery of patients.

Our study primarily demonstrated that evening preference, impaired sleep quality, and the severity of depression collectively exacerbate cognitive impairment in MDD. These findings, while requiring further validation in larger samples, underscore the clinical significance of understanding the neurobiological mechanisms underlying these associations. Chronobiological interventions and cognitive-behavioral therapy targeting sleep disturbances may offer promising avenues for improving cognition in MDD patients. Previous research has indicated that evening chronotype and poor sleep quality are common in MDD patients and are associated with cognitive dysfunction, social impairment, and lower quality of life (50). This suggests that clinicians should pay special attention to sleep-wake cycle disturbances when assessing cognitive function in MDD patients.

However, this study has certain limitations. The small sample size limited the robustness of our cognitive assessment and constrained the application of the usual three chronotypes classification analysis. Additionally, the cultural background of our sample, rooted in Chinese values of introversion, implicitness, and inclusiveness, may have influenced the way individuals self-regulate and absorb stress, potentially affecting our findings. Moreover, as a cross-sectional study, the data presented weak causal inferences, highlighting the need for future longitudinal studies to explore the connections between the remission of sleep disorders and cognitive impairment in MDD. A significant limitation is the lack of a control group composed of healthy volunteers, which restricts the ability to effectively compare cognitive function, chronotype preferences, and sleep quality. Furthermore, evidence suggests that cognitive deficits in MDD may persist even after mood symptoms improve, supporting the state, trait, and scar hypotheses (51). This further emphasizes the complexity of neurocognitive deficits in depression. Therefore, systematic identification and assessment of sleep and circadian preferences should be integral to the daily clinical management of depression, as this may help accelerate the functional recovery of patients. Finally, it may be necessary to consider the impact of pharmacological treatments and employ more specific measurement tools, such as polysomnography and circadian biomarker monitoring, to evaluate sleep effects in MDD patients more accurately.

This study highlights the prevalence of cognitive impairment in MDD patients and its close association with specific clinical factors, including first-episode status, severity of depression, circadian preference, and sleep quality. Additionally, our findings suggest that the co-existence of evening preference and poor sleep quality significantly increases the risk of cognitive impairment, highlighting these conditions as independent risk factors. These results emphasize the necessity for clinicians to pay special attention to circadian rhythms and sleep quality when assessing and treating patients with MDD. Targeted interventions addressing these factors could mitigate cognitive impairment in individuals suffering from major depression, advocating for a holistic treatment strategy that considers both the psychiatric and neurocognitive dimensions for improved patient outcomes.

## Data Availability

The original contributions presented in the study are included in the article/supplementary material. Further inquiries can be directed to the corresponding author.
